# Predictors of Multimorbidity Among Korean Older Adults: Longitudinal Secondary Data Analysis

**DOI:** 10.1093/geroni/igab046.626

**Published:** 2021-12-17

**Authors:** Eunkyung Kim, TaeWha Lee, Yoonjung Ji

**Affiliations:** Yonsei University, Seoul, Seoul-t'ukpyolsi, Republic of Korea

## Abstract

Multimorbidity has become a global concern for an aging society. It has been reported to be associated with increased health service utilization, leading to poor health outcomes including quality of life. However, the incidence of multimorbidity and its related factors are poorly understood. The aim of this study was to determine the socioeconomic and health-related factors predicting the incidence of multimorbidity in Korean older adults using longitudinal secondary data from the Korean Longitudinal Study of Aging (KLoSA) dataset from 2008 to 2018. The KLoSA aimed to collect basic data to be used for developing socioeconomic policy for the aging society in Korea. The sample included 3,019 older adults aged 65 years and over who had 0-2 chronic diseases at baseline in 2008. Multimorbidity was measured with the incidence of co-existence of three or more chronic diseases using Cox’s proportional-hazards model. Among 3,019 respondents (female 57.6%, mean age 73.07±6.30 years), 586 (19.4%) incidents of multimorbidity were reported after 10 years of follow-up. Low participation in social activities, being overweight or obesity, more depressive symptoms, current or past drinkers, and lower life satisfaction were identified as significant predictors of multimorbidity among Korean older adults. This study identified high risk groups with overlapping senility and multimorbidity, who require more attention from health care providers in the course of chronic disease monitoring and management. This longitudinal approach will contribute to the development of preventive strategies to reduce the incidence of multimorbidity among older adults.

